# A Possible Link between Food and Mood: Dietary Impact on Gut Microbiota and Behavior in BALB/c Mice

**DOI:** 10.1371/journal.pone.0103398

**Published:** 2014-08-18

**Authors:** Bettina Pyndt Jørgensen, Julie Torpe Hansen, Lukasz Krych, Christian Larsen, Anders Bue Klein, Dennis Sandris Nielsen, Knud Josefsen, Axel Kornerup Hansen, Dorte Bratbo Sørensen

**Affiliations:** 1 Section of Experimental Animal Models, Department of Veterinary Disease Biology, Faculty of Health and Medical Sciences, University of Copenhagen, Frederiksberg C, Denmark; 2 Neurobiology Research Unit, Rigshospitalet, Copenhagen, Denmark; 3 Department of Food Science, Faculty of Science, University of Copenhagen, Frederiksberg C, Denmark; 4 Bartholin Institute, Rigshospitalet, Copenhagen, Denmark; Charité-University Medicine Berlin, Germany

## Abstract

Major depressive disorder is a debilitating disease in the Western World. A western diet high in saturated fat and refined sugar seems to play an important part in disease development. Therefore, this study is aimed at investigating whether saturated fat or sucrose predisposes mice to develop behavioral symptoms which can be interpreted as depression-like, and the possible influence of the gut microbiota (GM) in this. Fourty-two mice were randomly assigned to one of three experimental diets, a high-fat, a high-sucrose or a control diet for thirteen weeks. Mice on high-fat diet gained more weight (p = 0.00009), displayed significantly less burrowing behavior than the control mice (p = 0.034), and showed decreased memory in the Morris water maze test compared to mice on high-sucrose diet (p = 0.031). Mice on high-sucrose diet burrowed less goal-oriented, showed greater latency to first bout of immobility in the forced swim test when compared to control mice (p = 0.039) and high-fat fed mice (p = 0.013), and displayed less anxiety than mice on high-fat diet in the triple test (p = 0.009). Behavioral changes were accompanied by a significant change in GM composition of mice fed a high-fat diet, while no difference between diet groups was observed for sucrose preferences, LPS, cholesterol, HbA1c, BDNF and the cytokines IL-1α, IL-1β, IL-6, IL-10, IL-12(p70), IL-17 and TNF-α. A series of correlations was found between GM, behavior, BDNF and inflammatory mediators. In conclusion, the study shows that dietary fat and sucrose affect behavior, sometimes in opposite directions, and suggests a possible association between GM and behavior.

## Introduction

Major depressive disorder (MDD) is a debilitating neuropsychiatric disease with high prevalence in the Western World population [1]. It is characterized by changes in behavior including e.g. anhedonia, anxiety, despair or hopelessness, decreased activities of daily living, poor concentration and decreased learning and memory abilities, as previously reviewed [2], [3]. A so-called western diet high in saturated fat and refined sugar, but low in omega-3 fatty acids, seems to play an important role in human disease development [4]. A possible linking factor between diet and depression may be the gut microbiota (GM), as diet has been shown to affect the composition of the GM [5], [6], and accumulating evidences indicate that the GM influences behavior [7]–[12]. Supporting this, MDD is often associated with a systemic low-grade inflammatory state [13], [14] and decreased brain neurogenesis [15], which both have been linked to the GM in rodent studies; Changes in the GM have previously been associated with increased levels of proinflammatory cytokines and behavioral changes [16]. Microbiota-induced stimulation of the immune system [17], [18], secondarily affecting behavior [16] may therefore be an important factor in development of MDD. Brain neurogenesis is highly dependent on brain-derived neurotrophic factor (BDNF), which is involved in learning and memory [19], and reported to be decreased in depressed patients [15], [20]. BDNF has been shown to be influenced by the GM, exemplified by changes in BDNF levels induced by germ-free conditions and fecal microbial transfer in BALB/c mice [21]. Based on these findings, it therefore seems plausible that the GM may be implicated in the association between diet and development of MDD. Previous rodent studies have partly investigated the relationship between diet and behavior, looking at links between diet and behavior [22], [23], GM and behavior [16], GM, neurochemistry and behavior [21], or diet, behavior and neurochemistry [24]. However, results are not consistent, demonstrating e.g. in one study that a high-calorie diet seems to cause neuroinflammation and depressive behavior [24], while in another study demonstrating that a high-calorie diet decreases depressive behavior and anxiety [23]. The reason for these discrepancies may be dietary differences regarding the contents of fat and sucrose; Macronutrients which may affect the GM, and subsequently the behavior, in different ways. In this study we therefore aimed to investigate whether the single dietary macronutrient saturated fat or sucrose predisposes mice for the development of behavioral symptoms which can be interpreted as MDD-like, and the possible mechanisms behind these changes. To date, no studies have investigated the association between diet, behavior, GM, inflammation, and neurogenesis in a single study, nor has a comprehensive investigation of the effect of the diet on the many aspects of depression-like behavior in rodents been performed. Both are important gaps which need to be filled in to fully understand the mechanisms of dietary impact on behavior.

Based on the previous studies of the GM and the well-documented link between the immune system and neuropsychiatric diseases [14], we therefore hypothesized that a diet-provoked change in GM composition could induce an imbalance within the local gut immune system, and increase the level of systemically circulating proinflammatory cytokines, thereby initiating neuroinflammation, resulting in behavioral changes of the mouse. We hypothesized that fat and sucrose would impact differently on the GM and subsequently on behavior, and therefore to investigate the single effect of fat and sucrose, the study was conducted subjecting mice to one of two experimental diets (high-fat/no-sucrose or high-sucrose/standard-low-fat diet) and evaluated by changes in GM composition, rodent behavior, metabolic markers, systemic low-grade inflammation, neuroinflammation and BDNF levels. The tests used to evaluate MDD-like behavior were the sucrose preference test assessing anhedonia, the burrowing test assessing species-specific behavior, the triple test assessing anxiety, the forced swim test (FST) assessing behavioral despair, and the Morris water maze assessing impairment in learning and memory abilities. The results obtained showed that fat and sucrose affect the GM and behavior differently. We found indications of an association between the GM and various aspects of behavior, with the immune system as a potential explanatory link.

### Materials and Methods

**Figure 1:**
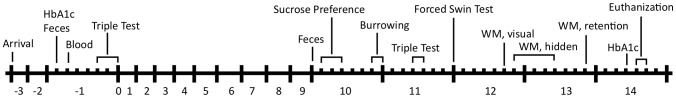
Timeline illustrating the experimental period. Numbers indicate week number, with mice subjected to the experimental diets from time 0. HbA1c: Glycosylated hemoglobin 1c, WM: Water Maze.

This study was conducted in strict accordance with the Council of Europe Convention European Treaty series (ETS) 123 on the Protection of Vertebrate Animals used for Experimental and Other Scientific purposes, and the Danish Animal Experimentation Act (LBK 1306 from 23/11/2007). The protocol was approved by the Animal Experimentation Expectorate under the Ministry of Justice, Denmark (License number 2012-15-2934-00256, C1). Mice were routineously checked on a daily basis, and efforts were made to minimize suffering and minimize the number of animals used. 42 male BALB/cAnNTac mice (Taconic, Denmark), at seven weeks of age, were specific pathogen free housed in standard polycarbonate cages with wire lid (type 1290, Tecniplast, Italy) equipped with Aspen bedding (Tapvei, Estonia), nesting material (Inviro-dri and Alpha-Nest, SSP, USA), a cardboard shelter (Sheperd's Shacks, SSP) and an Aspen gnawing block (Tapvei). During acclimatization, the mice had ad libitum access to tap water and standard rodent diet (Altromin 1324, Altromin, Germany). Temperature and relative humidity were 20–24°C and 55±10%, respectively, and the 12-hour light/dark cycle was shifted at 7 a.m. After two weeks of acclimatization, mice were randomly assigned to one of three diets, and fed either a high-fat/no-sucrose diet, a high-sucrose/standard low-fat diet or a control starch-based diet for nine weeks (all experimental diets were from Research Diets Inc., USA), see Table 1. The mice were housed pairwise until week five of the diet trial, then individually due to fighting. The mice were continued on their respective diets during behavioral testing. For a schematic overview of the timeline, see Figure 1.

**Table 1:** The experimental diets.

Diet	Control		High fat		High sugar	
Product#	D01060501		D0806104		D02022703	
	g%	kcal%	g%	kcal%	g%	kcal%
Protein	19.2	20	26.2	20	19.2	20
Carbohydrate	67.3	**70**	26.3	**20**	67.3	**70**
Fat	4.3	**10**	34.9	**60**	4.3	**10**
Total	90.8	100	87.5	100	90.8	100
kcal/g		3.85		5.24		3.85
	g	kcal	g	kcal	g	Kcal
Casein, lactic	200	800	200	800	200	800
L-cystine	3	12	3	12	3	12
Corn starch	575	2300	68.8	275	90	360
Maltodextrin 10	125	500	125	500	0	0
Sucrose	0	**0**	0	**0**	610	**2440**
Cellulose, BW200	50	0	50	0	50	0
Soybean oil	25	225	25	225	25	225
Lard	20	**180**	245	**2205**	20	**180**
Minerals and vitamins	Equal additions in all diets					

Differences between the diets are marked in bold.

Body weight and food intake were monitored weekly. Blood samples during the study were drawn by submandibular bleeding, and fecal samples were taken at relevant time points during the study (Figure 1). At euthanasia, the mice were anesthetized using a Hypnorm/Dormicum mixture 5 ml/kg (10 mg/ml fluanisone, 0.315 mg/ml fentanyl (VetaPharma, UK) and 5 mg/ml midazolam (Roche, Denmark)) before EDTA-stabilized blood and blood for serum preparation were drawn from the retro-orbital plexus, and centrifuged at 4000 *g* for 10 minutes and 10,000 *g* for four minutes, respectively. Fecal and cecal samples were kept on ice, whereas hippocampus and prefrontal cortex (PFC) were stored in liquid nitrogen before transfer to −80°C.

### 2.1 Behavioral testing

#### 2.1.1 The Sucrose Preference Test

The day before testing, the mice were habituated to a 2.5% sucrose solution for four hours. Subsequently, the mice were tested in a 24 h choice test, initiated at 11.00 a.m. with continuous access to food and two drinking bottles, one containing tap water, the other a 1% sucrose solution. The bottle position was switched after 12 hours, and the bottles were weighed before and after the test to calculate the amount of liquid consumed. All testing took place in the home cage of the mice.

#### 2.1.2 The Burrowing Test

Nesting material and shelters were replaced with a plastic tunnel (20 cm×diameter 7.2 cm) closed at one end, raised 5 cm at the other, and filled with 80 g of bedding material (Tapvei) for two hours between 3–5 p.m. in which period the mice were left undisturbed. Hereafter the remaining content of the tube was weighed, and the amount of bedding material burrowed out of the tube was calculated.

#### 2.1.3 The Triple Test

The test is a combination of three well-known anxiety tests, namely the open field (OF), the elevated plus maze (EPM) and the light/dark box (L/D), allowing exploring several aspects of anxiety without the tests interfering with each other [16], [25]. The dimensions of the apparatus are described in Fraser *et al.* (2010) [25]. The light intensities were 230–238 lx (OF, center), 35–60 lx (EPM, closed arms), 85–106 lx (EPM, open arms), 16–18 lx (LD, dark) and 1145–1270 lx (LD, light). The aversive zone of the OF was established as the center of the OF until ten centimeters from the outer walls. The mouse was placed in the center of the OF, and allowed to explore the maze for 6.5 minutes while video recorded, before it was returned to its cage. A cut-off value of 30 seconds to first move was used. The mice were subjected to this test twice; once prior to diet trial (pre-diet) and again after being subjected to the diet for nine weeks (post-diet).

#### 2.1.4 The Forced Swim Test

A conical glass cylinder (height 30 cm, diameter at water surface 12.5 cm) (Ikea, Denmark) was filled with room tempered water at a depth of 11 cm. The mouse was placed in the water for six minutes, and the behavior of the mouse was video recorded. After the test, the mouse was returned to its cage. Light intensity was 3–10 lx at the water surface.

#### 2.1.5 The Morris water maze test

A plastic pool (height 60 cm, diameter 120 cm) (Dansk Rotations Plast, Denmark) was filled with room tempered water at a depth of 15 cm, with a platform of clear plexiglass (diameter 10 cm) situated 1 cm below the surface. To teach the mice that they could escape the water by climbing the platform, a flag was placed on it to make it visible, and the mice were pre-trained by four swims of 60 seconds on day one with an intertrial interval of seven minutes. The mice were placed in the water at the same position for all four swims, with the platform placed at a different position each time. If the mouse found the platform, it was allowed to stay here for 15 seconds before it was returned to its cage. If not, it was picked up by the tail and placed on the platform for 15 seconds. The following five days the mice were given four trials of 60 seconds per day with a seven-minute inter-trial interval, starting from four positions different from those used during pre-training, and the hidden platform situated at a new and constant position. Three days after the last trial, the platform was removed, and all mice were given one 60 seconds retention swim trial, starting from a novel position. All trials were video recorded, and the time and distance used to reach the platform were measured.

### 2.2 Laboratory analysis

#### 2.2.1 Glycosylated hemoglobin A1c

To obtain information on whether the experimental diets induced hyperglycemia and metabolic stress in the mice, glycosylated hemoglobin A1c (HbA1c) was measured before the diet trial and the day before euthanasia by the use of the DCA Vantage Analyzer (Siemens, Denmark) and the associated DCA 2000 Hemoglobin A1c Reagent kit. Blood was obtained by tail vein puncture and the manufacturer's instructions were followed.

#### 2.2.2 Cytokines

Plasma and tissue samples were stored at −80°C until cytokine levels were measured by use of seven Mouse cytokine/chemokine FlowCytomix simplex kits (IL-1α, IL-1β, IL-6, IL-10, IL-12p70, IL-17 and TNF-α) (eBioscience, Austria). Tissue samples of hippocampus and PFC were weighed and homogenized in ice cold PBS buffer with 0.1% NP-40 (Sigma-Aldrich, Denmark), protease inhibitor tablets (Roche, Denmark) and 1 mM PMSF (Sigma-Aldrich), left for 20 minutes on ice, and centrifuged at 4 degrees at 10,000 *g* for 20 minutes before the supernatant was collected. Sample preparation was done by the plate method in accordance with the manufacturer's instructions, and bead fluorescence was measured by the use of BD FACSCanto II Flow cytometer (BD Biosciences, Denmark). Cytokine levels were calculated using the software Flowcytomix Pro 2.4 (eBioscience, Austria), and for tissue samples normalized to sample weight.

#### 2.2.3 BDNF

Tissue samples from hippocampus and PFC were homogenized in ice-cold RIPA buffer with 2 mM Na_3_VO_4_, 48 mM NaF and a protease inhibitor cocktail (Sigma-Aldrich, Denmark) by sonication 3×5 seconds on ice and centrifuged at 4 degrees at 10,000 *g* for 10 minutes before the supernatant was collected. The protein concentration was measured by the modified Lowry method (DC Protein Assay, Bio-Rad, Denmark). BDNF was measured by ELISA (Promega, Sweden) according to the manufacturer's instructions, and the absorbance was measured on an ELISA reader (MicroPlate Reader 550, Bio-Rad, Denmark). BDNF levels were normalized to the protein concentration in tissue samples.

#### 2.2.4 Lipopolysaccharide

Serum levels of the highly immunogenic bacterial lipopolysaccharide (LPS) were measured to determine whether the diet influenced the permeability of the gut, thereby initiating subchronic inflammation. Serum samples were analyzed using the PyroGene Recombinant Factor C Endotoxin Detection System (Lonza, Switzerland), following the manufacturer's instructions, with samples diluted 1∶100 and heated at 70°C for 10 minutes initially. Fluorescence was read on the SPECTRAmax GEMINI-XS plate reader (Molecular Devices, USA).

#### 2.2.5 Cholesterol

Total cholesterol was measured at euthanasia using the Accutrend Plus and Accutrend Cholesterol strips (Roche Diagnostics, North America), following the manufacturer's instructions.

#### 2.2.6 Denaturation gradient gel electrophoresis

Denaturation gradient gel electrophoresis (DGGE) was used to investigate differences in the composition of the GM. DNA was extracted from samples using the QIAamp DNA Stool Mini Kit (Qiagen, Germany). Fecal samples were dissolved in buffer by manual stirring followed by vortexing, and cecal samples disrupted by the FastPrep FP120 Cell Disrupter (QBiogen, MP Biomedicals, France, speed 5.5, 3×30 seconds). Hereafter the manufacturer's instructions were followed. Extracted DNA was stored at −20°C until PCR was performed. The PCR reaction mix consisted of five parts (out of 49) 10× DreamTaq Buffer (Fermentas, Thermo Fisher Scientific, USA), eight parts dNTP (1.25 mM, Bioline, Germany), one part each of the V3 region 16S rRNA gene targeting primers PRBA338fGC and PRUN518r (10 pmol/µl, Integrated DNA Technologies, USA and TAG Copenhagen, Denmark), 0.5 parts bovine serum albumin (5 ng/µl, New England Biolabs Inc., USA) and 0.5 parts DreamTaq DNA polymerase (Fermentas) mixed in 33 parts of MilliQ water. Extracted DNA was added to the mix using 47 µl of PCR mix to 3 µl of DNA sample in case of DNA from feces, or 49 µl to 1 µl DNA sample for cecal samples. The PCR reaction was run on a SureCycler 8800 (Agilent Technologies, USA), initialized by five minutes at 95°C followed by 33 repeated cycles of denaturing for 30 seconds at 95°C, annealing for 30 seconds at 60°C, and elongation for 45 seconds at 72°C, and a final step of 10 minutes of elongation at 72°C. Gels for DGGE were casted with 9% acrylamide and a denaturing gradient of formamide and urea increasing from 30% to 65% basically following the procedure described by Hufeldt *et al.* [26] although staining the gels for two hours.

#### 2.2.7 High throughput sequencing of the gut microbiota

The fecal (week 10) and cecal bacterial microbiota compositions were determined using tag-encoded 16S rRNA gene MiSeq-based (Illumina, CA, USA) high throughput sequencing. The V3-V4 region of the 16S rRNA gene was amplified using primers compatible with the Nextera Index Kit (Illumina) (NXt_V3-V4_F 5′-**TCGTCGGCAGC GTCAGATGTGTATAAG AGACAG**CCTAYGGGRB GCASCAG-3′ and NXt_V3-V4_R 5′-**GTCTCGTGGGCTCGGAGATGTGTATAAGAGACAG**GGACTACNNGGGTATCTAAT-3′; adapters in bold). PCR reactions containing 12 µl AccuPrime SuperMix II (Life Technologies, CA, USA), 0.5 µl of each primer (10 µM), 5 µl of genomic DNA (∼20 ng/ul), and nuclease-free water to a total volume of 20 µl were run on a SureCycler 8800 (Agilent, CA, USA). Cycling conditions applied were: Denaturation at 95°C for 2 min; 33 cycles of 95°C for 15 s, 55°C for 15s and 68°C for 40 s; followed by final elongation at 68°C for 5 min. To incorporate primers with adapters and indexes, PCR reactions contained 12 µl Phusion High-Fidelity PCR Master Mix (Thermo Fisher Scientific, USA, MA), 2 µl corresponding P5 and P7 primer (Nextera Index Kit), 2 µl PCR product and nuclease-free water for a total volume of 25 µl. Cycling conditions applied were: 98°C for 1 min; 12 cycles of 98°C for 10 s, 55°C for 20 s and 72°C for 20 s; elongation at 72°C for 5 min. The amplified fragments with adapters and tags were purified using AMPure XP beads (Beckman Coulter Genomic, CA, USA). Prior to library pooling clean constructs were quantified using a Qubit fluorometer (Invitrogen, Carlsbad, CA, USA) and mixed in approximately equal concentrations to ensure even representation of reads per sample followed 250 bp pair-ended MiSeq (Illumina) sequencing performed according to the instructions of the manufacturer.

### 2.3 Statistics

The triple test, forced swim test and Morris water maze test were analyzed using the software Ethovision vers. 5.0 (Noldus Information Technologies, The Netherlands). DGGE fingerprints were analyzed using the software Bionumerics vers. 4.5 (Applied Maths, Belgium) by principal component analysis (PCA) with a band position tolerance and optimization of 1%. The three primary components (PC1, 2 and 3) of the PCA were used to compare groups by ANOVA, as previously described [16]. For high throughput sequencing the raw dataset (NCBI accession number: SRP041490) containing pair-ended reads with corresponding quality scores was trimmed using CLC Genomic Workbench (CLC bio, Arhus, Denmark). Trimming settings were set to low quality limit of 0.01, with no ambiguous nucleotides allowed, and trimming off the primer sequences. Merging overlapped reads were performed using the “Merge overlapping pairs” tool using default settings. The Quantitative Insight Into Microbial Ecology (QIIME) tool (version. 1.7.0; Open source software) was used for further analysis [27]. Purging the dataset from chimeric reads was performed using USEARCH [28], while the Usearch61 method was used for Operational Taxonomic Units (OTU) selection [28]. The Greengenes (version 12.10) 16S rRNA gene database was used as a reference [29]. Principal coordinate analysis (PCoA) plots were generated with the Jackknifed Beta Diversity workflow based on 10 distance metrics calculated using 10 subsampled OTU tables. The -e value (number of sequences taken for each jackknifed subset) was set to 85% of the sequence number within the most indigent sample. Samples whose number of reads was below 50,000 and 40,000 for fecal and cecal samples, respectively, were removed from this step. Analysis of similarities (ANOSIM) was used to evaluate group differences using weighted and unweighted uniFrac distance metrics that were generated based on rarefied (50,000 and 40,00 reads per sample or fecal and cecal samples respectively) OTU tables. The relative distribution of the genera registered was calculated for unified and summarized in the genus level OTU tables. Alpha diversity measures expressed with an observed species (sequence similarity 97% OTUs) value were computed for rarefied OTU tables (50,000 and 40,000 reads per sample or fecal and cecal samples, respectively) using the alpha rarefaction workflow. Differences in alpha diversity were determined using a t-test-based approach employing the non-parametric (Monte Carlo) method (999 permutations) implemented in the compare alpha diversity workflow. The G test of independence (q_test) and ANOVA were used to determine: Qualitative (presence/absence) and quantitative (relative abundance) association of OTUs with given diet. These were calculated based on 1000 subsampled OTU-tables rarefied to an equal number of reads (50,000 and 40,000 reads per sample or fecal and cecal samples, respectively). Both the *p*-value and the conservative FDR-corrected *p*-value for multiple comparisons are reported. 3D plots were constructed from the three primary PCs from the PCoA of the MiSeq analysis to visualize group differences in the composition of the GM. Statistics were processed in R (The R foundation for statistical computing, Austria) or SAS JMP vers. 10.3 (SAS Institute Inc., US). In general, adherences to normality distribution were checked by QQ plots and the Goodness of fit test. Means and standard errors or medians are reported when relevant. A *p*-value<0.05 was considered significant. Groups were compared using one-way ANOVA with the Tukey honest significance test correction for *post hoc* analysis when data were normally distributed, and when not by the non-parametric Kruskal-Walis Test followed by the Wilcoxon each pair *post hoc* correction for multiple comparisons. The Morris water maze test was analyzed by ranking all 20 trials for each mouse and using the repeated measurement two-way ANOVA. Simple linear regression was used to investigate the association between levels of BDNF and plasma cytokines and behavior, and multiple linear regression models were created using the three primary PCs from the PCoA of the MiSeq analysis (as previously described [16]) to investigate the relationship between GM and behavior, inflammatory mediators and BDNF. Linear models were validated using the QQ plots of residuals and predicted values/residuals plots, and robustness of significance was tested by removing a random single sample from the dataset twice.

## Results

### 3.1 Body weight and food intake

No difference in body weight was evident between the groups at arrival (p = 0.95), but after one week on the experimental diets, the mice on high-fat diet were significantly heavier than the two other groups (p = 0.00009). This significant difference persisted all through the study period, with the average weight in groups reaching 32.79±0.66 g, 29.02±0.38 g and 28.28±0.53 g for the high-fat, control and high-sucrose diet group, respectively. However, the weight was not correlated to performance in any behavioral test or levels of cytokines or BDNF (Table 2), neither did the mice on high-fat diet move a shorter distance in the triple test (median 1010 cm, 990 cm and 1098 cm for high-fat, control and high-sucrose diet group, respectively, p = 0.55), and the swim speed in the Morris water maze was similar to the other groups (13.0±1.0 cm/s, 12.5±1.2 cm/s and 13.0±1.5 cm/s for high-fat, control and high-sucrose diet group, respectively, p = 0.21), indicating that the weight itself did not influence performance in the behavioral tests.

**Table 2:** Multiple linear regression analysis between GM and behavior, cytokines, and BDNF.

Location	Diet group	PCoA Analysis	Principal component	Anhedonia		Species-typical behavior		Anxiety				Coping behavior in the FST				Memory		Hippocampal BDNF		Systemic inflammatory markers							
								Time spent in LB of LDB		Time spent on OA		Latency to immobility		Time spent immobile						IL-6		IL-12p70		IL-17A		IL-1α	
				*p*	r^2^	*p*	r^2^	*p*	r^2^	*p*	r^2^	*p*	r^2^	*p*	r^2^	*p*	r^2^	*p*	r^2^	*p*	r^2^	*p*	r^2^	*p*	r^2^	*p*	r^2^
**Body weight**			-		-		-		-		-		-		-		-		-		-		-		-		
**Feces**	All	UW	PC1	-		*0.027*	*0.24*	-		-		*0.039*	*0.12*	-		*0.029*	*0.22*	-		**0.049**	**0.15**	-		-		-	
			PC2	-		-		-		-		-		-		*0.0051*	*0.22*	-		-		-		-		-	
			PC3	-		*0.032*	*0.24*	-		-		-		-		-		-		-		-		-		-	
		W	PC1	-		**0.025**	**0.14**	-		-		-		-		**0.035**	**0.12**	-		*0.015*	*0.22*	-		-		-	
			PC2	-		-		-		-		0.021	0.15	-		-		-		-		-		-		-	
	Control	UW	PC2	-		-		-		-		-		-		*0.029*	*0.56*	*0.02*	*0.42*	-		-		-		-	
		W	PC2	-		-		-		-		-		-		-		**0.0027**	**0.61**	-		-		-		-	
			PC3	-		-		-		*0.0036*	*0.29*	-		-		-		-		-		-		-		-	
	High-sucrose	UW	PC1	-		-		-		-		-		-		-		-		-		-		-		0.019	0.72
			PC2	-		-		-		-		-		-		*0.056*	*0.35*	-		-		-		-		0.004	0.72
			PC3	-		*0.0049*	*0.60*	-		-		-		*0.016*	*0.53*	-		-		-		-		-		-	
		W	PC3	-		-		-		-		-		*0.018*	*0.52*	-		-		-		-		-		-	
	High-fat	W	PC3	*0.025*	*0.41*	-		-		-		-		-		-		-		*0.041*	*0.47*	-		-		-	
**Cecum**	All	UW	PC3	-		-		*0.026*	*0.14*	-		-		-		-		-		-		-		-		-	
		W	PC1	-		-		-		-		-		-		-		-		**0.036**	**0.16**	-		-		-	
	Control	UW	PC1	-		-		-		-		-		-		-		-		-		**0.0084**	**0.97**	-		-	
			PC2	-		-		-		-		-		-		-		-		-		**0.0042**	**0.97**	-		-	
			PC3	-		-		-		-		-		-		**0.046**	**0.34**	-		-		*0.0039*	*0.97*	-		-	
		W	PC2	-		-		-		-		-		-		*0.0061*	*0.59*	-		-		-		-		-	
	High-sucrose	UW	PC2	-		-		-		-		-		-		-		-		-		-		*0.011*	*0.82*	-	
		W	PC2	-		-		-		-		-		-		-		-		**0.036**	**0.65**	-		-		-	
			PC3	-		-		-		-		-		-		-		-		-		-		**0.051**	**0.44**	-	
	High-fat	UW	PC1	-		-		-		-		-		-		-		-		-		-		**0.058**	**0.67**	-	
			PC2	-		-		-		-		-		-		-		-		-		-		**0.028**	**0.67**	-	
		W	PC1	**0.016**	**0.49**	-		-		-		-		-		-		-		-		*0.028*	*0.47*	-		-	

Several significant associations are seen between GM and both behavior and systemically circulating inflammatory mediators, indicating a possible influence from the gut on the immune system and the brain. Furthermore a certain GM composition seems to be associated with both improved memory and increased levels of BDNF in mice not subjected to any of the experimental diets. The PCoA analysis denotes whether data was weighted (W) or not (UW) by bacterial abundance and the principal component (PC) listed denotes the significant factor(s) in the multiple linear regression models. Not shown and non-significant are: Risk assessment on OA, Time spent in OF center and PFC BDNF. GM was not correlated to IL-1β, IL-10 and TNF-α, as all samples were at basal concentrations regarding these cytokines. *Italic: negative correlation*. **Bold: Positive correlation**.

### 3.2 Behavioral tests

#### 3.2.1 Sucrose Preference Test

No differences in sucrose preference were found in relation to diet, neither for the relative amount of sucrose solution consumed (median 65.22%, 68.02% and 63.89% for high-fat, control and high-sucrose diet group, respectively, p = 0.94) nor for the absolute amount of sucrose solution (median 1.8 g, 1.9 g and 2.0 g for high-fat, control and high-sucrose diet group, respectively, p = 0.98), indicating that the diet itself did not induce profound anhedonic-like behavior.

#### 3.2.2 Burrowing Test

**Figure 2:**
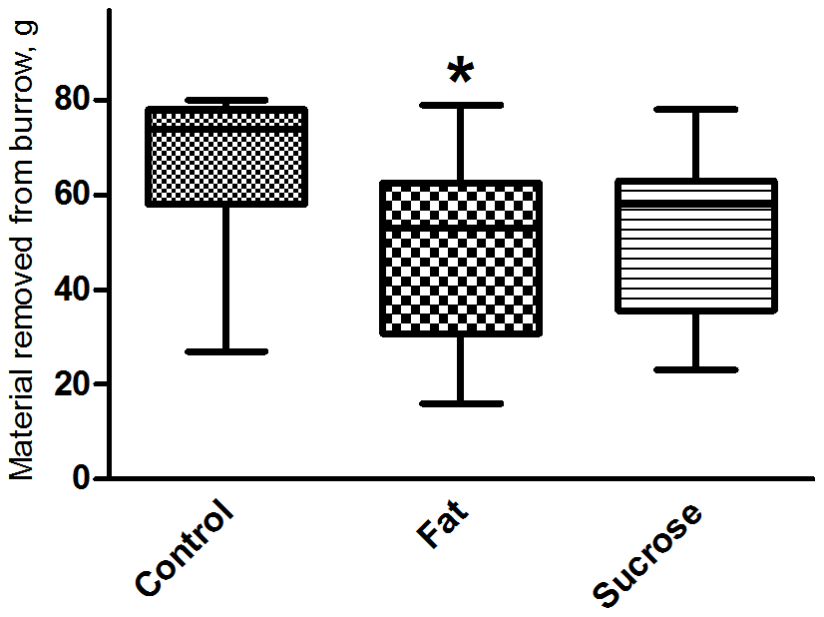
The Burrowing test. Mice on HF diet removed significantly less bedding material from the tube than mice on C diet (p = 0.035). A tendency of reduced burrow-digging is seen for mice on HS diet (p = 0.064). However, although not quantified, this diet group burrowed less goal-oriented, as observations showed excessive digging in the whole cage. Median with ranges.

Mice on high-fat diet burrowed significantly less bedding out of the tube than mice on control diet (median 53 g and 74 g respectively, p = 0.034) (Figure 2). A similar reduced burrowing, although not statistically significant, was observed in the high-sucrose group (median 58 g, p = 0.064). Notably, observations during the test revealed that mice on high-sucrose diet showed more sporadic digging behavior than the other groups, with a substantial part of the behavior directed towards the cage bedding, hence not only confining the digging to the tube.

#### 3.2.3 Triple Test

**Figure 3:**
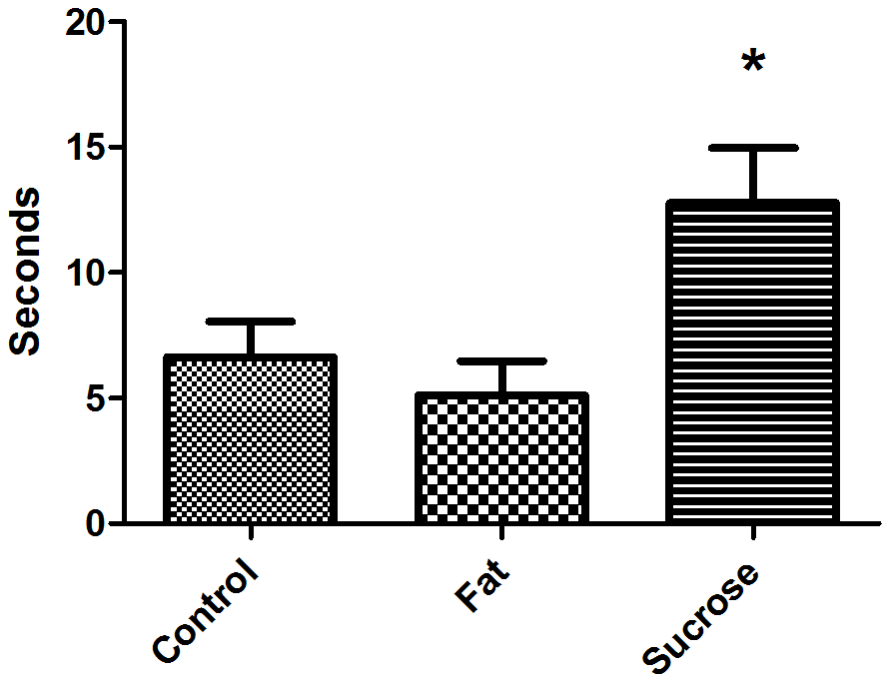
The Triple test, time spent in the aversive centre of the open field at post-diet test. Mice on high-sucrose diet spent significantly more time here than the mice on high-fat diet, indicating a decreased anxiety (p = 0.009). A strong tendency of a similar difference to mice on control diet supports this decreased anxiety in mice fed a high-sucrose diet (p = 0.052). Mean with SE.

Four mice were eliminated from the test as they failed to move within 30 seconds. No difference was found between diet groups for proportion of time spent in either OF, EPM or L/D box neither pre-diet (median 15%, 25% and 19%, p = 0.25, 77%, 65% and 70%, p = 0.57 and 10%, 11% and 10%, p = 0.97 for high-fat, control and high-sucrose diet group respectively) nor post-diet (median 20%, 22% and 23%, p = 0.79, 71%, 70% and 68%, p = 0.92 and 15%, 12% and 14%, p = 0.33 for high-fat, control and high-sucrose diet group, respectively). This was also the case for the proportion of time spent at open arms of EPM (pre-diet testing median 0%, 2% and 1,8%, p = 0.50 for high-fat, control and high-sucrose diet group, respectively, and post-diet testing median 0%, 0% and 0%, p = 0.93 for high-fat, control and high-sucrose diet group, respectively), and proportion of time spent in the light department of L/D box (pre-diet testing median 0%, 0% and 0%, p = 0.93 for high-fat, control and high-sucrose diet group, respectively, and post-diet testing median 5.1%, 4% and 3%, p = 0.85 for high-fat, control and high-sucrose diet group, respectively) and proportion of time spent in center of the OF at pre-diet testing (median 1.2%, 2.5% and 4%, p = 0.13 for high-fat, control and high-sucrose diet group, respectively). However, at post-diet testing, mice on sucrose diet displayed less anxiety than the other diet groups as they spent significantly more time in the aversive center of the OF than mice on high-fat diet (mean 3.2±0.55%, total time 12.8±2.19 sec. and 1.3±0.34%, total time 5.1±1.36 sec., p = 0.009) and displayed a strong tendency of a similar reduced anxiety when compared to the control group (mean 1.7±0.35%, total time 6.6±1.40 sec., p = 0.052) (Figure 3).

#### 3.2.4 Forced swim test

Mice on high-sucrose diet displayed significantly increased latency to immobility compared to both the control group (p = 0.039) and the high-fat diet group (p = 0.013) (median 83 sec., 59 sec. and 53 sec. For high-sucrose, control and high-fat diet group, respectively), which may indicate hyperactive behavior. No difference was seen between diet groups in the duration of immobility (194±11.81 sec., 176.80±20.54 sec., and 172±19.13 sec. for the high-fat, control and high-sucrose diet group, respectively, p = 0.66).

#### 3.2.5 Morris water maze

**Figure 4:**
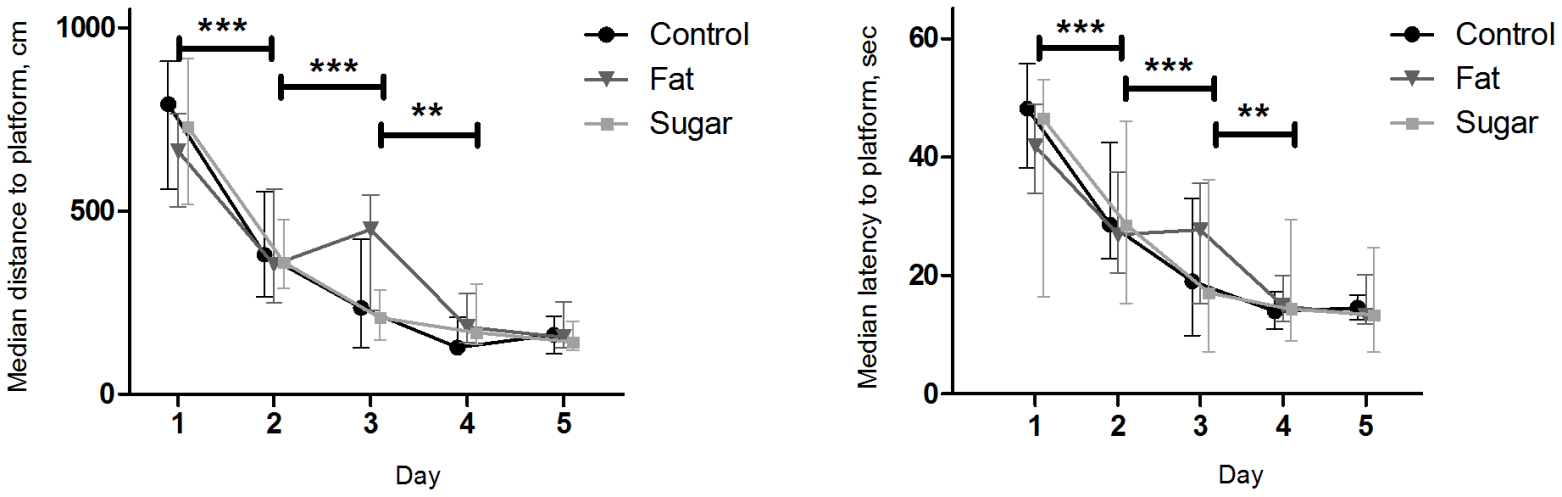
The Morris water maze test, distance swum and latency to reach the platform. A general day to day significant decrease in distance swum and latency to reach platform was seen for all diet groups, indicating that all diet groups learned the task. However, a high-fat diet seemed to influence negatively on coping with the start position sequence on day three. Median with ranges.

*Hidden platform*: All mice learned the test, indicated by an overall significant day-to-day decrease in both distance swum to the platform and latency to reach the platform observed from day one to four (p<0.001–0.01 and p<0.001–0.05 for distance and latency, respectively), while no improvement was seen from day four to five (p = 0.87 and p = 0.91 for distance and latency, respectively) (Figure 4). These decreases in distance and latency were unaffected by diet (p = 0.62 and p = 0.38 for distance and latency, respectively). However, mice on high-fat diet seemed to have more difficulties coping with the start-position sequence at day three than mice fed high-sugar or control diet, reflecting that even though the mice learned the task, a high-fat diet may impair cognitive functioning (median distance 451 cm, 237 cm and 210 cm for high-fat, control and high-sucrose diet group, respectively, p = 0.10 and median latency to reach platform 28 sec., 19 sec. and 17 sec., p = 0.22 for high-fat, control and high-sucrose diet group, respectively).

**Figure 5:**
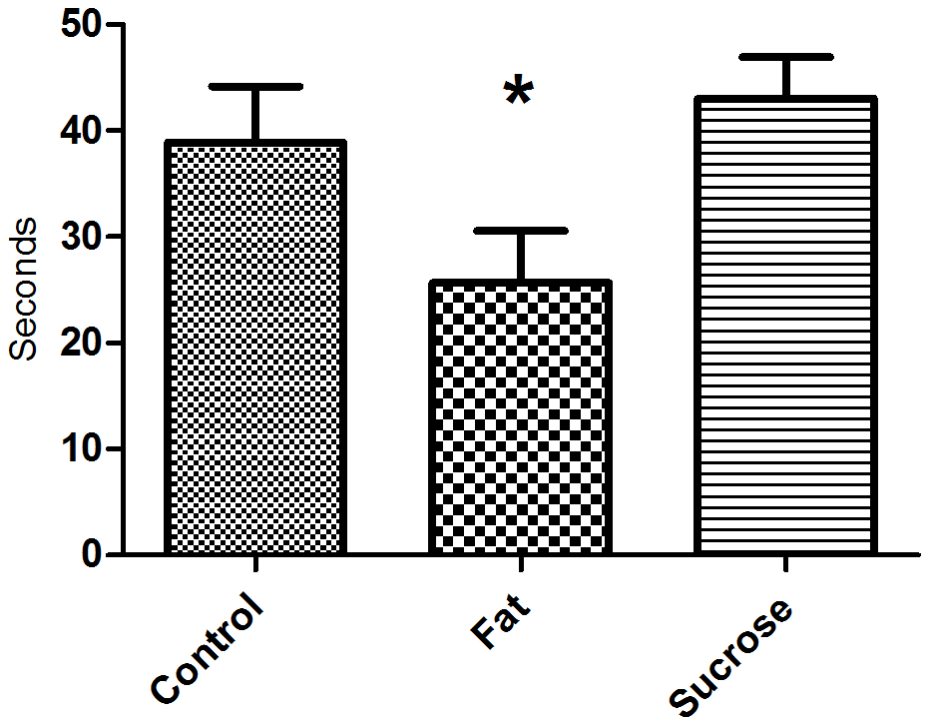
The Morris water maze test, preference for the previous platform area. Mice on high-fat diet spent significant less time in the area of the previous situated platform during the first 30 seconds of the retention trial compared to mice on high-sucrose diet. Memory of mice on control diet was similar to mice on high-sucrose diet. This indicate, that consuming a high-fat diet leads to decreased memory. Mean with SE.

*Retention test*: Memory was affected by diet, as the mice on high-fat diet showed significantly lower preference for the area surrounding the previous platform than mice on high-sucrose diet during the first 30 seconds (26%±4.80 vs. 43%±3.93, p = 0.031) (Figure 5). Area preference in the control group was 38%±5.28%. This indicates different dietary effects on memory performance, with a high-fat diet negatively influencing memory.

#### 3.3.1 Biochemical analyses

Diet did not affect long-term blood glucose of the mice, as the HbA1c levels did not differ between the groups at baseline (mean 3.2±0.032, p = 0.38) nor at the end of the experiment (mean 3.3±0.021, p = 0.28). Cholesterol levels were near detection limit, with no difference between groups (median 3.98, 3.93 and 3.90 mmol/L for the high-fat, control and high-sucrose diet groups, respectively). No difference in serum LPS-levels was detected between groups (median 52.50, 57.25 and 59.00 endotoxin units/ml for high-fat, and control and high-sucrose diet group, respectively, p = 0.43).

BDNF were measured in the brain regions hippocampus and prefrontal cortex involved in the behavioral processes of the tests. At the time of euthanasia no difference in BDNF concentration was found in relation to diet, neither in hippocampus (median 161.30, 168.70 and 155.90 pg/mg protein for high-fat, control and high-sucrose diet group, respectively, p = 0.73) nor in PFC (median 66.44, 73.41 and 72.12 pg/mg protein for high-fat, control and high-sucrose diet group, respectively, p = 0.27). The diet groups did not differ with regard to inflammation, measured by cytokine levels in plasma and the brain regions PFC and hippocampus. The levels of the measured cytokines were in general low, indicating no diet-induced profound systemic inflammation (Table 3).

**Table 3:** Cytokine concentrations in plasma, prefrontal cortex and hippocampus.

Cytokine	Diet group	Plasma			PFC			Hippocampus		
		*Min*	*Median*	*Max*	*Min*	*Median*	*Max*	*Min*	*Median*	*Max*
**IL-1α**	High-fat	*<dl*	67.36	75.78	*<dl*	1.64	4.82	*<dl*	3.77	5.4
	Control	*<dl*	68.56	130.53	*<dl*	1.92	5.27	*<dl*	4.3	7.64
	High-sucrose	*<dl*	66.25	81.6	*<dl*	1.27	5.48	*<dl*	2.8	5.27
**IL-6**	High-fat	*<dl*	11.06	39.84	*<dl*	*<dl*	1.45	*<dl*	2.19	11.16
	Control	*<dl*	8.82	23.58	*<dl*	*<dl*	1.68	*<dl*	4.3	9.59
	High-sucrose	*<dl*	8.56	23.81	*<dl*	*<dl*	2.26	*<dl*	2.1	7.25
**IL-10**	High-fat	*<dl*	*<dl*	109.74	*<dl*	*<dl*	7.35	*<dl*	4.12	20.56
	Control	*<dl*	*<dl*	45.38	*<dl*	*<dl*	12.51	*<dl*	5.6	25.51
	High-sucrose	*<dl*	*<dl*	18.75	*<dl*	*<dl*	16.35	*<dl*	6.35	27.15
**IL-1β**	High-fat	*<dl*	*<dl*	56.75	*<dl*	*<dl*	3.89	*<dl*	2.93	6.79
	Control	*<dl*	*<dl*	*<dl*	*<dl*	*<dl*	1.36	*<dl*	2.94	14.97
	High-sucrose	*<dl*	*<dl*	27.87	*<dl*	*<dl*	3.85	*<dl*	1.64	10.5
**IL-12p70**	High-fat	*<dl*	10.45	23.41	*<dl*	2.12	6.29	*<dl*	3.69	10.64
	Control	*<dl*	5.46	19.96	*<dl*	*<dl*	5.77	*<dl*	5.48	20.79
	High-sucrose	*<dl*	6.26	25.31	*<dl*	2.86	6.71	*<dl*	2.74	17.26
**TNF-α**	High-fat	*<dl*	2.34	89	*<dl*	*<dl*	1.04	*<dl*	*<dl*	*<dl*
	Control	*<dl*	*<dl*	14.15	*<dl*	*<dl*	2.66	*<dl*	*<dl*	*<dl*
	High-sucrose	*<dl*	*<dl*	22.19	*<dl*	*<dl*	1.25	*<dl*	*<dl*	*<dl*
**IL-17A**	High-fat	*<dl*	9.47	41.78	*<dl*	*<dl*	1.7	*<dl*	*<dl*	*<dl*
	Control	*<dl*	3.01	22.06	*<dl*	*<dl*	1.77	*<dl*	*<dl*	*<dl*
	High-sucrose	*<dl*	7.82	27.17	*<dl*	*<dl*	1.79	*<dl*	*<dl*	*<dl*

Median, minimum and maximum levels in each diet group are given, measured in pg/ml plasma or pg/mg brain tissue. No differences in inflammatory state are observed between groups, and levels are in general subclinically, which was also expected. Particular IL-1β, IL-10 and TNF-α are at very low concentrations. Min: Minimum, max: Maximum. *<dl*: Below detection limit.

#### 3.3.2 Associations between inflammatory markers or BDNF and behavior

The low-grade levels of the systemic inflammatory mediators IL-6, IL-12p70 and IL-17A correlated to memory, anxiety, anhedonia and species-typical behavior (Table 4), indicating a possible influence on behavior. IL-6 was negatively correlated with memory. High levels of IL-12p70 were associated with decreased sucrose consumption, decreased species-typical behavior, increased anxiety (measured by increased numbers of peaks into the light box, without entering, and decreased time spent in the light box), and decreased memory functioning, whereas IL-17A was found to correlate positively with increased sucrose consumption and good memory. No significant linear regressions were observed between behavior and BDNF measurement.

**Table 4:** Associations between cytokine levels in plasma and behavior as determined by linear regression analyses.

Cytokine	Diet group	Anhedonia		Species-typical behavior		Anxiety				Memory	
						Peaks into LB of LDB		Time spent in LB of LDB			
		*p*	r^2^	*p*	r^2^	*p*	r^2^	*p*	r^2^	*p*	r^2^
**IL-6**	All									*0.015*	*0.21*
**IL-12p70**	All	*0.015*	*0.23*								
	Control							*0.011*	*0.83*		
	High-sucrose			*0.004*	*0.77*					*0.034*	*0.55*
	High-fat					**0.030**	**0.42**				
**IL-17A**	All	**0.057**	**0.14**								
	High-sucrose									**0.034**	**0.55**

Several associations were found between systemically circulating inflammatory markers and behavior. Statistically significant linear regressions revealed that high levels of IL-6 was related to poor memory performance, and high levels of IL-12p70 increased anxiety in the triple test, measured by increased numbers of peaks into the light box, without entering, and decreased time spent in the light box. IL-17A is associated with amount of sucrose consumption and memory. Behavior was not correlated to IL-1β, IL-10 and TNF-α, as all samples were at basal concentrations regarding these cytokines. Not shown and non-significant are: In the triple test; Time spent in center of OF, time spent on OA, risk assesment to OA, and in the FST; Latency to and time spent immobile. *Italic: negative correlation*. **Bold: Positive correlation**.

### 3.4 Composition of the gut microbiota

**Figure 6:**
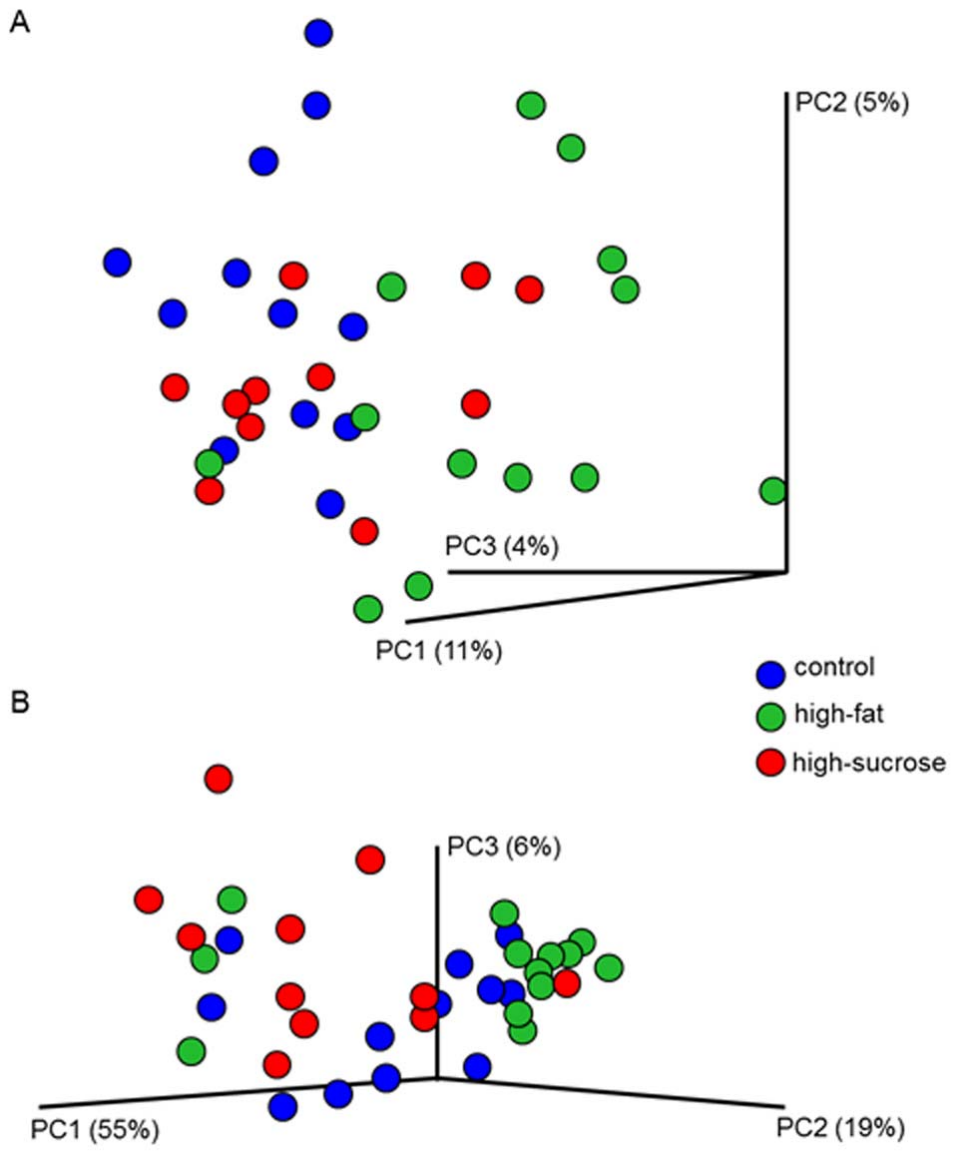
3D plots of the unweighted and weighted PCoA of cecum. Plots are constructed from the three most primary principal components of the PCoA, with A) showing the unweighted analysis and B) showing the weighted analysis which takes the abundance of the bacteria into account. Both plots visualize that the cecal GM of mice fed a high-fat diet diverge from that of mice fed either a high-sucrose or a control diet.

Based on PCA of the DGGE analysis, no difference was observed between groups before diet trial (p = 0.54, 0.67 and 0.12 for PC1, PC2 and PC3 respectively)., but after nine weeks on the experimental diets, the GM of mice on high-fat diet differed significantly from the GM of mice on the control diet (PC2, p = 0.028) and the GM of mice on high-sucrose diet (PC2, p = 0.041), indicating an effect of diet on GM (See the Figures S1 and S2 for a dendrogram of the DGGE fingerprints from week 10 and the boxplot of PC2). No difference was found between mice on high-sucrose and control diets (p = 0.98). High throughput sequencing yielded 2,346,983 sequences free from chimeric reads, providing an average of 106,681 sequences per sample (minimum = 34,137; maximum = 177,160; SD = 40,228) with a mean sequence length of 432 bp (SD = 14 bp). ANOSIM of the sequencing results confirmed that the fecal GM of mice on high-fat diet differed significantly from that of mice on both control (unweighted, p = 0.004, R = 0.25) and high-sucrose diet (unweighted, p = 0.028, R = 0.15), while the GM of mice on high-sucrose diet did not differ significantly from that of mice on control diet (unweighted, p = 0.11, R = 0.070). Taken into account the abundance of the bacteria, the same picture was seen (weighted, high-fat vs. control, p = 0.055, R = 0.12, high-fat vs. high-sucrose p = 0.047, R = 0.14, control vs. high-sucrose p = 0.61, R = −0.027). The Firmicutes phylum was significantly increased in feces of high-fat fed mice (36.0%, 20.1% and 17.6%, p = 0.0061, FDR p = 0.024 for high-fat, control and high-sucrose diet group, respectively), primarily within the families Rumunococcaceae and Lachnospiraceae, and especially within the genus *Ruminococcus* (1,32%, 0.71% and 0.48%, for high-fat, control and high-sucrose diet group, respectively, p = 0.0087, FDR p = 0.049, table 5). The Bacteroidetes phylum decreased in feces of high-fat fed mice (37.3%, 55.8% and 60.0%, p = 0.045, FDR p = 0.090 for high-fat, control and high-sucrose diet group, respectively), with a significant reduction of an unclassified genus belonging to the family S24-7 (0.45%, 1.68% and 1.52%, p = 0.00027, FDR p = 0.006, Table 4), resulting in a decreased Bacteroidetes/Firmicutes (B/F) ratio in the high-fat diet group (median 0.94, 3.30 and 4.53 for high-fat, control and high-sucrose diet group, respectively, high-fat vs. high-sucrose p = 0.024, high-fat vs. C p = 0.087). The analysis of the cecal GM revealed the same picture as in feces, showing a significant difference between mice fed high-fat and control diets (unweighted, p = 0.002, R = 0.17) and high-fat and high-sucrose diets (unweighted, p = 0.006, R = 0.18), but not between mice fed high-sucrose and control diet (unweighted, p = 0.11, R = 0.070). Taking the relative abundance of the different UOT's into account revealed the same tendency (weighted, high-fat vs. control, p = 0.088, R = 0.060, high-fat vs. high-sucrose p = 0.062, R = 0.076, control vs. high-sucrose p = 0.14, R = 0.046). 3D plots constructed from the three primary PC's of the unweighted and weighted PCoA visualized the results (cecum, figure 6A and B. Feces, not shown): The GM of mice fed a high-fat diet diverged from the GM of mice fed a high-sucrose or a starch-rich control diet in the PCoA plot constructed from unweighted data (Figure 6A), and taken into account the bacterial abundance, the same tendency was seen (Figure 6B). The Firmicutes phylum also increased in cecum of the high-fat fed mice (37.7%, 24.1% and 22.4% p = 0.0062, FDR p = 0.056, for high-fat, control and high-sucrose diet group, respectively), primarily within the family Ruminococcaceae (20.31%, 13,.65% and 12.16%, p = 0.00062, FDR p = 0.014, table 6), whereas the Bacteroidetes phylum decreased near-significantly in the high-fat group (17.0%, 26.4% and 23.8%, p = 0.052, FDR p = 0.12 for high-fat, control and high-sucrose diet group, respectively), primarily within an unclassified genus belonging to the family S24-7 (0.14%, 0.34% and 0.29%, p = 0.025, FDR p = 0.19, for high-fat, control and high-sucrose respectively, table 5), resulting in a significantly reduced B/F ratio in the high-fat fed mice, (median 0.51, 0.88 and 1.13 for high-fat, control and high-sucrose diet group, respectively, high-fat vs. control p = 0.0036, high-fat vs. high-sucrose p = 0.0021, high-sucrose vs. control p = 0.68). G tests revealed no diet-induced difference in presence or absence of bacterial species in neither feces nor cecum.

**Table 5:** Dietary-induced differences in fecal microbial composition at week 10.

Phylum	Community	Order	Family	Genus	*p* value	FDR corrected	High-fat	High-sucrose	Control
**Tenericutes**	Mollicutes	Anaeroplasmatales	Anaeroplasmataceae	*Anaeroplasma*	0,0001	0,0057	0,0005	0,0044	0,0473
**Bacteroidetes**	Bacteroidia	Bacteroidales	S24-7	*Unassigned*	**0,0003**	**0,0060**	**0,4496**	**1,5243**	**1,6772**
**Firmcutes**	Clostridia	Clostridiales	Ruminococcaceae	*Oscillospira*	0,0052	0,0779	7,3167	3,1129	2,9530
**Firmcutes**	Clostridia	Clostridiales	Peptpcoccaceae	*Unassigned*	0,0055	0,0624	0,0157	0,0031	0,0047
**Firmcutes**	Clostridia	Clostridiales	Unassigned	*Unassigned*	0,0061	0,0553	0,2468	0,1039	0,1122
**Firmcutes**	Clostridia	Clostridiales	Lachnospiraceae	*Dorea*	0,0073	0,0547	0,1082	0,0510	0,0341
**Firmcutes**	Clostridia	Clostridiales	Lachnospiraceae	*Unassigned*	0,0083	0,0533	3,6198	1,5299	1,4754
**Firmcutes**	Clostridia	Clostridiales	Ruminococcaceae	*Ruminococcus*	**0,0087**	**0,0492**	**1,3186**	**0,4843**	**0,7131**
**Firmcutes**	Clostridia	Clostridiales	Ruminococcaceae	*Unassigned*	0,0103	0,0513	0,1471	0,0501	0,0601
**Firmcutes**	Clostridia	Clostridiales	Lachnospiraceae	*Coprococcus*	0,0150	0,0676	0,7503	0,3797	0,2803
**Firmcutes**	Clostridia	Clostridiales	Unassigned	*Unassigned*	0,0249	0,1017	16,8028	8,3336	11,5668
**Bacteroidetes**	Bacteroidia	Bacteroidales	Unassigned	*Unassigned*	0,0250	0,0936	0,0362	0,0897	0,1485
**Proteobacteria**	Alphaproteobacteria	Unassigned	Unassigned	*Unassigned*	0,0282	0,0976	0,0014	0,0021	0,0070
**Firmcutes**	Clostridia	Clostridiales	Lachnospiraceae	*(Ruminococcus)*	0,0371	0,1193	0,5036	0,2784	0,2619

The table shows bacterial OTU's which differs with a significant *p*-value between diet groups. A high-fat diet impacts the GM composition, significantly increasing the abundance of Firmicutes, primarily within the families Ruminococcaceae and Lachnospiraceae, and significantly reducing the abundance of an unclassified genus of the family S24-7 within the Bacteoidetes phylum. *P*-values and the FDR-corrected *p*-values are listed.

**Table 6:** Dietary-induced differences in cecal microbial composition.

Phylum	Community	Order	Family	Genus	*p* value	FDR corrected	High-fat	High-sucrose	Control
**Firmicutes**	Clostridia	Clostidiales	Ruminococcaceae	*Unassigned*	**0,0001**	**0,0023**	**3,0084**	**1,6692**	**1,9505**
**Firmicutes**	Clostridia	Clostidiales	Ruminococcaceae	*Oscillispira*	**0,0006**	**0,0139**	**17,3053**	**10,4943**	**11,6951**
**Firmicutes**	Unassigned	Unassigned	Unassigned	*Unassigned*	0,0055	0,0822	0,0829	0,0467	0,0600
**Firmicutes**	Clostridia	Unassigned	Unassigned	*Unassigned*	0,0131	0,1469	5,1159	2,5009	2,7778
**Firmicutes**	Clostridia	Clostidiales	Unassigned	*Unassigned*	0,0132	0,1186	1,2720	0,6663	0,8006
**Bacteroidetes**	Bacteroidia	Bacteroidales	S24-7	*Unassigned*	0,0253	0,1900	0,1354	0,2865	0,3362
**Cyanobacteria**	4C0d-2	YS2	Unassigned	*Unassigned*	0,0276	0,1772	0,0214	0,0332	0,0770
**Bacteroidetes**	Bacteroidia	Bacteroidales	Prevotellaceae	*Prevotella*	0,0312	0,1754	0,0018	0,0025	0,0085
**TM7**	TM7-3	CW040	F16	*Unassigned*	0,0312	0,1561	0,0049	0,0062	0,0169
**Firmicutes**	Clostridia	Clostidiales	Ruminococcaceae	*Ruminococcus*	0,0372	0,1672	1,1954	0,8930	0,6906
**Firmicutes**	Clostridia	Coriobacteriales	*Unassigned*	*Unassigned*	0,0451	0,1846	0,0257	0,0084	0,0104

The table shows bacterial OTU's which differs with a significant *p*-value between diet groups. A high-fat diet impacts the GM composition, significantly increasing the abundance of Firmicutes, primarily within the family Ruminococcaceae, and reducing the abundance of the family S24-7 within the Bacteoidetes phylum. *P*-values and the FDR-corrected *p*-values are listed.

### 3.5 Associations between gut microbiota, behavior, inflammation and brain neurogenesis

Multiple linear regression analyses revealed an extensive association between the GM composition and behavior. Based on high throughput sequencing we found significant associations between GM and anhedonia, species-typical behavior, anxiety, coping behavior in an inescapable environment, and memory (Table 6). Furthermore, associations were found between GM and systemically levels of the proinflammatory cytokines IL-1α, IL-6, IL-12p70 and IL-17A, indicating an influence of the GM on the immune system (Table 6). For the control group, which was not challenged by an experimental diet, fecal GM composition was found to correlate with both hippocampal levels of BDNF and memory performance in the Morris water maze (Table 6), with mice having a distinct GM composition having high levels of BDNF and good memory performance. These multiple associations between GM composition and behavior and inflammatory mediators were supported by significant linear relationships between abundance of specific bacteria of the phylum Bacteroidetes to memory performance and a marker of low-grade inflammation; In feces increased abundance of an unclassified genus belonging to the S24-7 family correlated to better memory performance (p = 0.00045 FDR p = 0.010, r^2^ = 0.55), and an increased abundance of the genus *Bacteroides* correlated significantly to lower levels of plasma IL-6, although not after FDR correction (p = 0.029 FDR p = 1.29, r^2^ = −0.37), but nevertheless suggesting a positive influence of these bacteria. In cecum increased amount of a bacteria of an unassigned genus in the Bacteroidales order correlated significantly to better memory performance, although not when the conservative FDR correction was applied (p = 0.0047, FDR p = 0.21, r^2^ = 0.45), this suggests a relationship, and supports the observations in feces.

## Discussion

### 4.1 Dietary effects on behavior

The present study shows that the dietary components saturated fat and sucrose affect behavior of BALB/c mice. Furthermore, for some types of behavior, e.g. memory, anxiety and coping strategies, the individual effect of fat and sucrose on behavior seems to be opposite to each other, with one enhancing and one impairing the specific type of behavior.

Consuming a high-fat diet led to significantly less species-specific burrowing behavior compared to a control diet. Furthermore, a high-fat diet significantly affected memory capabilities in the Morris water maze negatively compared to a high-sucrose diet, and although not statistically significant, mice on high-fat diet displayed difficulties coping with the start position sequence on day three of the learning phase. These observations are in accordance with a previous study using an experimental diet containing both fat and sucrose or sugar [19], and our study suggests that the deficits previously reported may be ascribed to the fat content of the diet. In summary, as both the burrowing test and the Morris water maze test are hippocampal-dependent, this indicates that dietary saturated fat interferes with hippocampal functioning and affects behavior influenced by this brain area.

A diet high in sucrose also affected species-specific burrowing behavior, with mice displaying a strong tendency of decreased goal-oriented digging behavior compared to mice on control diet. However, mice on this diet were observed to dig vigorously sporadic places within the cage during the test, thus creating a very bumpy bedding, indicating that a diet high in sucrose impacts on the goal-oriented part of this test, and not on the burrowing behavior itself. Unfortunately, the digging outside the tube could not be quantified, and therefore this remains to be investigated further. In the FST mice fed a high-sucrose diet stayed mobile for significantly longer before displaying the first period of immobile floating compared to both the high-fat and control group. Notably, despite the initial increased endurance, this group did not float less than the other groups during the six-minute test. Despite the high predictive validity of the FST in tests of antidepressants, this test has been heavily debated for whether it resembles despair, or whether it instead reflects different coping strategies in an inescapable environment [30]. The present study points to the latter, with the diet influencing the coping strategy. HbA1c levels were not increased in the high-sucrose fed group, indicating a good metabolic glucose control of the mice. However, we cannot exclude the possibility that mice fed a high-sucrose diet possessed a larger glycogen-storage in the liver and muscles, and were therefore capable of displaying an increased initial endurance in the FST test. The triple test revealed significantly decreased anxiety to an open area in the sucrose-fed mice compared to mice fed a high-fat diet, with a similar strong tendency when compared to control mice. This cannot be explained by hyperactivity, as all diet groups traveled the same distance in the non-aversive zones of the test. A previous study using a diet high in both fat and sugar reported decreased anxiety in rat dams subjected to maternal separation [23]. Based on the results of the present study, we propose that the reported effect was due to the sugar content rather than the fat content of the diet. In summary, the present study indicates that a high-sucrose diet affects coping strategies, possibly through increased endurance, decreases anxiety, and causes compromised abilities in goal-oriented tasks.

Although we did see behavioral changes in the BALB/c mice on the experimental diets, they did not develop profound MDD-like symptoms. When comparing our results with previous studies, the combination of an unhealthy high-calorie diet *and* a genetically sensitive background and/or severe stress seems to have greater impact on rodent behavior, than we achieved with only the diet itself [22], [23], [31]. Noteworthy, there may be a synergistic effect of dietary fat and sugar when combined in a diet. We demonstrate that they affect the GM, the body and the mind in different ways, and thus a possible synergistic effect of fat and sucrose on behavior may likely depend on the relative amount of these within a specific diet.

### 4.2 Analysis of the gut microbiota

Consuming a high-fat diet significantly changed the GM in both feces and cecum, which is in accordance with previous studies comparing the GM of mice fed a high-fat or a starch-rich diet [5]. An altered GM has previously been associated with changes in learning and memory abilities [12], and anxiety and exploratory behavior [8], [16], [21]. A study by P. Bercik (2011) demonstrated that fecal microbial transfer of the GM from BALB/c mice to NIH Swiss mice and vice versa resulted in a behavioral phenotype related to the donor-strain when mice were tested for anxiety and exploratory behavior, clearly revealing an effect of the GM on behavior [21]. Likewise, offspring of mice subjected to the maternal immune activation (MIA) autism spectrum model has been shown to display a significantly different GM profile and elevated plasma levels of the bacterial metabolite 4-ethylphenylsulfate, which when administered to naïve mice induces behavioral changes [32]. Therefore, it cannot be rejected that the observed high-fat diet induced shift in the GM may have contributed in mediating the behavioral changes observed. The GM of mice fed high-sucrose and control diet did not differ significantly in feces or cecum. However, simple carbohydrates, such as sucrose, are metabolized in the small intestine. A recent work by B. van den Bogert (2013) showed variable carbohydrate fermentation capacities and distinct immunomodulatory characteristics among the different streptococcal strains situated in the small intestine [33], [34], and reported fluctuations in bacterial composition of the small intestine to be diet-related [35]. Based on this it therefore seems likely that the GM of the small intestine was modulated by diet in mice receiving a high-sucrose diet, which may secondly have affected behavior. Further investigations into the dietary impact on the GM of the small intestine and whether or not a specific diet-induced shift in the GM affects behavior, would be needed to conclude further on the observations in both dietary treatment groups.

### 4.3 Analysis of inflammatory markers, BDNF and metabolic markers

In order to exclude some of the metabolic parameters known to affect behavior [36]–[38], we measured total cholesterol and long-term blood glucose. These parameters did not differ between diet groups, and therefore we conclude that they had no impact on behavior in the present study. As we aimed at investigating diet-induced effects on the GM and subsequently on behavior, the choice of mouse strain and the length of the diet trial were chosen to avoid the metabolic effect of long term feeding, and the obtained results support this. The gut permeability was not compromised by the diets, as the serum levels of the highly immunogenic bacterial LPS were similar in all diet groups. Elevated levels of LPS in the bloodstream is usually linked to an inflamed and compromised leaky gut, as this is the main reservoir of gram-negative bacteria in the body, and a high-fat diet has previously been associated with elevated levels of systemic LPS and inflammation [6], [39]. Similar systemic LPS levels among diet groups are on the other hand in agreement with the present findings of no difference between diet groups regarding inflammation. BDNF levels were similar across diet groups. However, a learning task may increase the BDNF synthesis [40], and therefore the short time span from the Morris water maze to euthanasia may have diminished a difference between the groups.

### 4.4 Associations between GM, behavior, BDNF and inflammatory markers

We showed significant associations between GM and anxiety, anhedonia, species-specific behavior, coping behavior, memory, and inflammatory mediators. In the control group, which was not challenged by an experimental diet, the GM composition was furthermore correlated on the same principal component to both memory and hippocampal BDNF levels, with the latter known to affect memory, supporting an influence of the GM on memory. Based on this wide association found between the GM and the many aspects of behavior, we suggest a general influence of the GM on the gut-brain-axis (GBA) through one or several mechanisms, of which the present study supports that the immune system may be one. We found the GM composition to be associated with systemic levels of the proinflammatory cytokines IL-12p70 and IL-17A, which are produced by dendritic cells and Th17 cells situated in the gut epithelium in response to bacterial stimulation. The low-grade levels of these systemically circulating inflammatory markers were secondly significantly associated with behavior; We found levels of IL-6, IL-12p70 and IL-17A to significantly correlate with memory, anxiety, anhedonia and species-typical behavior. This suggests that these cytokines are used as signaling molecules, and supports the hypothesis, that the GM may influence behavior through modulation of the immune system.

Many previously reported detrimental effects of a high-fat diet may be ascribed to a diet-induced decrease in assumable “good and protective” bacteria. Supporting this, antibiotic or probiotic treatment of rodents on a high-fat diet has shown to affect cholesterol- and triglyceride levels [41], improve glucose tolerance [42], and improve memory and anxiety-related behavior [43]. In the present study a high-fat diet reduced the abundance of an unclassified genus from the family *S24-7*, of which high abundance was significantly correlated to good memory performance and showed tendencies of being associated with lower levels of the inflammatory mediator IL-6. Such single bacterial correlations, however, would need confirmation from additional studies. Nevertheless, the GM of mice fed a high-fat diet also correlated to sucrose preference, a measure of anhedonic behavior. These results suggest a fat-induced dysbalance in the GM composition may contribute in making the individual prone to develop symptoms of depression-like behavior.

In summary, the study demonstrates differentiated dietary impact on behavior and shows correlations between the GM, behavior and the immune system. The observed behavioral changes may be unrelated to the GM, but rather mediated by dietary-induced metabolic or hormonal mechanisms not investigated in the present study, and the observed associations between GM and behavior may be a result of the bidirectional GBA, with the brain affecting the gut, secondly affecting the GM composition. However, the correlations found between GM and the inflammatory mediators, and between the GM-related inflammatory mediators IL-12p70 and IL-17a and behavior suggest an impact of the GM on behavior, possibly through the immune system, disregarding diet and the host's influence on the GM. Furthermore, a significant diet-induced reduction of a genus from the S24-7 family, of which increased abundance correlated to good memory performance, suggests a diet-induced impact of the GM on behavior. Therefore, it cannot be rejected that the GM contributes in affecting behavior, and the observed behavioral changes may very likely be an outcome of the combination of several mechanisms affecting the brain, such as metabolic, hormonal and microbial. Further studies of diet trials in germ-free mice and of mice subjected to microbial transfer of diet-modulated microbiota from the different sections of the intestine needs to be performed, in order to evaluate more on the role of the GM in the relationship between diet and behavior. However, using germ-free mice or controlling a transferred microbiota limits the choice of behavioral tests, as tests lasting more than one day, e.g. cognition assessing tasks like the Morris water maze, may not be evaluated by such studies.

## Conclusions

Based on the negatively affected memory, the impaired species-specific behavior, which is thought to reflect capability of human daily activities, the diet-induced change in GM and the association between GM and anhedonic-like behavior in mice receiving a high-fat diet, our results suggests that a diet high in saturated fat contributes to development of depression-like behavior, and that changes in the GM may be considered a mediator. Levels of LPS, cholesterol, HbA1c, cytokines or BDNF could not explain the observed diet-induced behavior. The effect of a high-sucrose diet on behavior may be mediated through other metabolic pathways, as we saw no significant change in the GM of feces and cecum in this diet group. However, it cannot be excluded, that the high-sucrose diet impacted the GM of the small intestine, secondly modulating the immune system and behavior. Importantly, we show that fat and sucrose affect behavior differently and sometimes oppositely, and thus the proportion of fat and sugar in the diet should be paid more interest when designing behavioral studies. Finally, we documented a wide association between the GM, behavior, BDNF, and the immune system, and although not stating causality, the present study emphasizes the need for more research into the impact of the GM on behavior both in general and in disease.

## Supporting Information

Figure S1 Dendrogram of the cluster analysis based on fecal DGGE fingerprints of week 10. The boxes on the right show the clustering at 74% similarity level (blue line). As it is seen, seven mice on high-fat diet show strong similarity in their GM, despite being housed individually. Some clustering is also seen for the two other groups, visualized by colored boxes containing animals from only one diet group. A: f = high-fat diet, c = control diet, s = high-sugar diet. B: The DGGE-gel the sample was run on. C: Mouse number.
(TIF)

Figure S2 Boxplot showing the difference in fecal microbial composition at week 10 of the diet trial. The second principal component of the principal component analysis based on the DGGE fingerprints showed that diet influence the gut microbiota composition as mice consuming a high-fat diet differ significantly in GM composition from the mice on sucrose diet (p = 0.041) and control diet (p = 0.028) after 9 weeks on the experimental diets.
(TIF)
