# Supplementary figures and images for: A Possible Link between Food and Mood: Dietary Impact on Gut Microbiota and Behavior in BALB/c Mice
Source: PLoS One. 2014 Aug 18;9(8):e103398. doi: 10.1371/journal.pone.0103398 (PMC4136797; doi:10.1371/journal.pone.0103398)

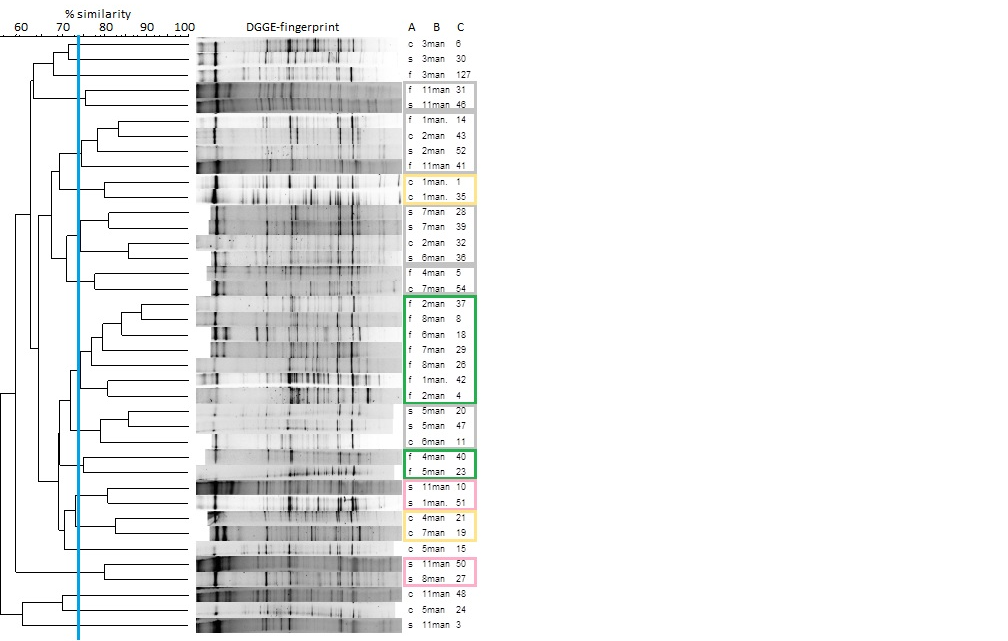

Supplement: Figure S1 — Dendrogram of the cluster analysis based on fecal DGGE fingerprints of week 10. The boxes on the right show the clustering at 74% similarity level (blue line). As it is seen, seven mice on high-fat diet show strong similarity in their GM, despite being housed individually. Some clustering is also seen for the two other groups, visualized by colored boxes containing animals from only one diet group. A: f = high-fat diet, c = control diet, s = high-sugar diet. B: The DGGE-gel the sample was run on. C: Mouse number. (TIF) [file pone.0103398.s001.tif]

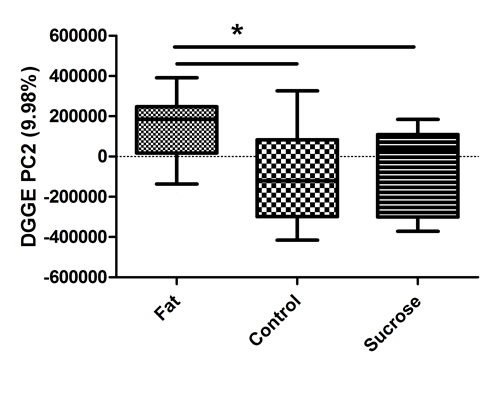

Supplement: Figure S2 — Boxplot showing the difference in fecal microbial composition at week 10 of the diet trial. The second principal component of the principal component analysis based on the DGGE fingerprints showed that diet influence the gut microbiota composition as mice consuming a high-fat diet differ significantly in GM composition from the mice on sucrose diet (p = 0.041) and control diet (p = 0.028) after 9 weeks on the experimental diets. (TIF) [file pone.0103398.s002.tif]
